# The Eye, Nose and Throat

**Published:** 1902-09

**Authors:** 


THE EYE, NOSE AND THROAT. Vol. III of the Practical Medicine Series of Year Books, comprising ten volumes of the year's progress in medicine and surgery. Under the general editorial charge of Gustavus P. Head, M. D., Professor of Laryngology and Rhinology, Chicago Post-Graduate Medical School. Edited by Casey A. Wood, C. M., M. D.; Albert H. Andrews, M. D.; T. M. Hardie, M. D. Pages, 346. Price per volume, $1.50, or $7.50 for whole series. Chicago: The Year Book Publishers, 40 Dearborn St. December, 1901.

These little books have been coming out monthly, and contain very satisfactory summaries of medical progress, each in the line of its particular subject. T. J. B.

